# Identification and *In Vitro* Functional Verification of Two Novel Mutations of *GHR* Gene in the Chinese Children with Laron Syndrome

**DOI:** 10.3389/fendo.2021.605736

**Published:** 2021-04-12

**Authors:** Ran Li, Fengying Gong, Hui Pan, Hanting Liang, Hui Miao, Yuxing Zhao, Lian Duan, Hongbo Yang, Linjie Wang, Shi Chen, Huijuan Zhu

**Affiliations:** Key Laboratory of Endocrinology of National Health Commission, Department of Endocrinology, Peking Union Medical College Hospital, Chinese Academy of Medical Science and Peking Union Medical College, Beijing, China

**Keywords:** Laron syndrome, *GHR* gene mutation, subcellular distribution, STAT5, HepG2 cells

## Abstract

**Purpose:**

Laron syndrome (LS) is a severe growth disorder caused by *GHR* gene mutation or post-receptor pathways defect. The clinical features of these patients collected in our present study were summarized, *GHR* gene variants were investigated and further *in vitro* functional verification was carried out.

**Methods:**

Four patients with LS were collected, their clinical characteristics were summarized, genomic DNA was extracted, and *GHR* gene was amplified and sequenced. GHR wild type (GHR-WT) and mutant GHR expression plasmids were constructed, and transiently transfected into HepG2 cells and HEK293T cells to observe the subcellular distribution of the GHR protein by immunofluorescence and to determine the expression of GHR and its post-receptor signaling pathway changes by Western blotting.

**Results:**

All of the four patients were male, and the median height was -4.72 SDS. Four *GHR* gene variants including c.587A>C (p.Y196S), c.766C>T (p.Q256*), c.808A>G (p.I270V) and c.1707-1710del (p.E570Afs*30) were identified, and the latter two were novel mutations. The results of mutant GHR plasmids transfection experiments and immunofluorescence assay showed that the subcellular distribution of GHR-Q256* and GHR-E570Afs*30 mutant proteins in HepG2 and HEK293T cells presented with a unique ring-like pattern, gathering around the nucleus, while GHR-Y196S mutant protein was evenly distributed on HepG2 cell membrane similar to GHR-WT. The GHR protein levels of HepG2 cells transiently transfected with GHR-Y196S, GHR-Q256* and GHR-E570Afs*30 were all significantly lower when compared with cells transfected with GHR-WT (P<0.05). Further mutant GHR post-receptor signal transduction investigation demonstrated that GH induced phosphorylated STAT5 levels of HepG2 cells transfected with three mutant plasmids were all significantly decreased in comparison with that of GHR-WT (P<0.05).

**Conclusions:**

Two novel *GHR* gene mutations (I270V and E570Afs*30) were found in our patients with LS. GHR mutations influenced the subcellular distribution and GHR protein levels, then led to the impaired post-receptor signal transduction, suggesting that the *GHR* mutations contributed to the pathological condition of LS patients.

## Introduction

Laron syndrome (LS) is a rare inherited disorder characterized by severe postnatal growth failure, normal or increased circulating growth hormone (GH) secretion and insulin-like growth factor 1 (IGF-1) deficiency (1). Classical LS was first described by Laron et al. in 1966 who reported three siblings with severe growth retardation from a consanguineous Jewish family, and the disorder was termed “Laron-type dwarfism” or “growth hormone insensitivity” subsequently (2, 3). LS is a kind of autosomal recessive disease which often occurred in families with parental consanguinity and sporadic cases have also been reported (3). Since 1966 when LS was firstly reported, more than 250 patients have been reported so far (4, 5).

LS is mainly caused by growth hormone receptor (GHR) gene mutations and monogenic defects of post-receptor components in the GH signal transduction pathway, such as signal transducer and activator of transcription 5B (*STAT5B*), *IGFALS*, *IGF1*, *IGF-1R* and pregnancy-associated plasma protease A2 (*PAPPA2*) (5–13). Among the various genetic aberrations, *GHR* gene mutations are most commonly reported (4).

GH, also known as somatotropin, plays a critical role in the promotion of growth, cell division and regeneration (3, 4). The effects of GH are directly mediated through its cell surface receptor, GHR. *GHR* gene is located on chromosome 5p13.1-p12 and is composed of 10 exons, with exon 1 being an untranslated region (3, 14). Exons 2 to 10 encode a peptide of 638 amino acid residues. After GHR proteins are synthesized in the endoplasmic reticulum, they are transported to the cell membrane through a protein-conducting channel and inserted in the cell membrane (15, 16). The mature GHR protein is comprised of three domains: an extracellular domain of 246 residues (encoded by exons 2-7 of GHR), a transmembrane domain of 24 residues (encoded by exon 8), and an intracellular domain of 350 residues (encoded by exons 9 and 10) (17). GH binds with GHRs to form a trimolecular complex and changes the conformation of the GHRs, triggers signaling molecules, such as Janus kinase 2 (JAK2), STAT5, and Src family kinases (2, 18–20). Activated, tyrosine-phosphorylated STAT5 proteins translocate into the nucleus and promote the transcription of the downstream IGF-1 gene (6, 21).

To date, more than 100 *GHR* gene defects have been identified, and the majority of the mutations occur in the extracellular domain of *GHR* gene, while mutations in the transmembrane domain and intracellular domain are relatively rare (4, 17, 22, 23). Several studies investigated the effects of *GHR* gene variations on the GH post-receptor signal transduction *in vitro* experiments. Fang et al. reported a compound heterozygous *GHR* mutations, C94S and H150Q, in an Austrian family, and functional studies showed that both the compound heterozygous mutation and C94S heterozygous mutation resulted in deficiency in activating GH-induced gene expression and diminished GH-induced STAT5b activation (24). Rughani et al. reported a novel W267* heterozygous nonsense mutation in the transmembrane domain in a Caucasian child, and the W267* mutation was shown to inhibit GH-induced STAT5 activation (25). Besides missense and nonsense mutation, the c.784G>C splicing mutation was investigated in a Turkish child, and *in vitro* studies revealed that the c.784G>C splicing mutation destroyed the intron 7 donor site and led to an absence of functional GHR (26). While, intragenic GHR deletion of 1454 nucleotides led to exon 8 skipping from the GHR mRNA transcript and translated a truncated GHR protein (27).

In the present study, four Chinese children diagnosed with LS in our Department of Endocrinology were collected and their clinical and biochemical characteristics were summarized. *GHR* gene variants were investigated and further *in vitro* functional verification was performed by cellular experiments to further elucidate the effects of *GHR* gene variations on *GHR* gene expression, subcellular distribution and GH-induced signal transduction.

## Materials and Methods

### Clinical and Biochemical Characteristics

Four Chinese patients with severe growth retardation were admitted to Peking Union Medical College Hospital from 2012 to 2017. Height and weight were recorded as standard deviation scores (SDSs) based on the age- and sex-appropriate reference ranges of Chinese children. Blood samples were collected in the morning after an overnight fast, and the concentrations of GH and IGF-1 were measured by a solid-phase, two-site, chemiluminescent immunometric assay (IMMULITE 2000, Siemens, UK). Blood biochemical parameters and whole blood cell count were measured. The L-dopa GH provocative test was conducted as follows: L-dopa was given at a dosage of 125mg to patients weighing less than 10 kilograms (kg), 250 mg for patients weighing 10-30 kg, and for patients weighing more than 30 kg, 500 mg L-dopa was given (28). The basal GH level was quantified before medication, and peripheral serum was sampled every 30 minutes for up to 2 hours to measure GH concentration. The insulin-induced hypoglycemia GH provocative test was carried out by subcutaneously injecting insulin at a dosage of 0.1 U/kg, and the time-point of blood collection was 0, 30, 60, 90 and 120 minutes after medication (29). A peak GH concentration after stimulation of <5 ng/ml was used to diagnose growth hormone deficiency (28). Pituitary magnetic resonance imaging (MRI) was conducted in each patient. LS was suspected when patients presented with extreme postnatal growth failure and midfacial hypoplasia. Biochemically, LS was featured by normal or elevated GH secretion and subnormal serum IGF-1 concentration, demonstrating the inability to generate normal quantities of IGF-1. *GHR* gene sequencing was then performed for patients suspected of LS, and the detailed process was described in the following part. The parents of the patients have given their written informed consent. The study was approved by the Ethics Committee of Peking Union Medical College Hospital and the reference number was JS-1663.

### DNA Extraction and *GHR* Gene Sequencing

Genomic DNA was extracted from peripheral blood leukocytes using the Qiagen DNeasy Blood Kit (Qiagen, 69504, Germany) according to the standard protocol. Coding exons (exon 2 to exon 10) and the boundaries between introns and exons of the *GHR* gene were amplified by polymerase chain reaction (PCR) method. The primers used for PCR amplification were shown in **Table 1**. All PCR products were sequenced (Tianyi Huiyuan Biotech Corporation, Beijing, China) and aligned with the standard *GHR* sequence (RefSeq NM_000163.5) in UCSC BLAT (http://genome.ucsc.edu/cgi-bin/hgBlat). Our sequencing data is uploading to GenBank, and the accession numbers are MW701347, MW701348 and MW701349.

**Table 1:** Primers used in the PCR amplification for *GHR* gene.

Exon	Forward primer sequence	Reverse primer sequence	Length of PCR products
Exon 2	AGCTCATTCATGTCTTACCC	AAAACTTGGATGTAGCGAAT	252bp
Exon 3	AGCCACAAAATGACCTGTTTAGC	GCCACACACTTTTAAACAACCAGA	781bp
Exon 4	CTAGACACGGAATACACTGG	AACAAATCACTTCCATTCCCACA	347bp
Exon 5	GAAGTACCAAACGGCCTC	TCTTCTTCACAACATTTACTGC	611bp
Exon 6	AAAATATTGGAAGAAATAAGAGCA	GGCCTCCATATATACATAAGCATC	516bp
Exon 7	AAAATGGGAGAATACCTG	ATATTTTGATTTGGACAACAC	349bp
Exon 8	GCTGAAACCTTTATGATACTCCC	CTGGAATGAATGGGTCAACT	758bp
Exon 9	ACACTCCAATTATATAAAGTACCA	TCCAGGAGAAGAGACACAAG	331bp
Exon 10	ACTGTTGTTCTTATTGTAACCAT	AAGGCATTTTAGAATCCATACCC	1213bp

### Construction of the *GHR* Wild-Type and Mutant Expression Plasmids

The full-length human *GHR* gene cDNA (NM_000163.5) was obtained by RT-PCR method from human total RNA isolated from white blood cells using EZNA Total RNA Kit (Omega Bio-Tek, Doraville, USA). GHR wild-type (GHR-WT) expression plasmid was yielded by inserting the *GHR* gene whole cDNA (+193~+2109) into pcDNA3.1(+) vector, both *GHR* gene cDNA and the pcDNA3.1(+) vector were digested by Kpnl and EcoRI enzymes and were ligated by T4 DNA ligase (Thermo Fisher Scientific, USA). The HA tag sequence (TACCCCTACGACGTGCCCGACTACGCC) was inserted after the signal peptide sequence, then the plasmid was sequenced. Site-directed mutagenesis experiments were performed by Vigene Biosciences Company (Beijing, China) to construct GHR-Y196S, GHR-Q256* and GHR-E570Afs*30 mutant expression plasmids and Sanger sequencing was performed to verify the *GHR* gene mutations. Expression plasmids were extracted with a Qiagen Plasmid Maxi Kit (Qiagen 12162, Germany).

### Cell Culture

Human embryonic kidney (HEK293T) cells and HepG2 cells were purchased from Cell Resource Center of Institute of Basic Medicine, Chinese Academy of Medical Sciences. HEK293T cells were cultured in DMEM medium (Hyclone, USA) supplemented with 10% fetal bovine serum (Cell Resource Center of Institute of Basic Medicine, Beijing, China) and antibiotics (100U/ml penicillin and 100U/ml streptomycin from Gibco, USA) under 5% CO_2_ and 95% O_2_ at 37°C. HepG2 cells were maintained in MEM medium (Hyclone, USA) containing 10% fetal bovine serum (Cell Resource Center of Institute of Basic Medicine, Beijing, China), nonessential amino acids (Gibco, USA) and antibiotics.

### Transient Transfection

HEK293T cells and HepG2 cells were seeded on 12-well plates at a density of 6×10^5^ cells/well. The cells were grown to 50~60% confluence prior to removal of antibiotic-containing medium and then incubated with 1ml antibiotic-free medium per well. The cells were transfected with 1.0 μg expression plasmids (GHR-WT, GHR-Y196S, GHR-Q256* and GHR-E570Afs*30 expression plasmids) and 1.5 μl of Lipofectamine 3000 (Thermo Fisher Scientific, USA) in 100 μl of OPTI-MEM serum-free medium (Hyclone, USA) for 5 hours, then the culture medium was replaced with fresh medium containing 10% fetal calf serum for 48h. The vector group was transfected with 1.0 μg pcDNA3.1(+) empty vector instead of expression plasmids.

### Immunofluorescence Assays

Immunofluorescence was performed on fixed cells to determine the subcellular distribution of GHR. HEK293T cells and HepG2 cells were plated on 12-well plates treated with polylysine and transfected with either GHR-WT or mutant GHR expression plasmids. 48 hours after transfection, the cells were fixed with 4% paraformaldehyde for 20 minutes and permeabilized with saponin (Beyotime Biotechnology, P0095, China) for 20 minutes. QuickBlockTM blocking solution (Beyotime Biotechnology, P0220, China) was added to block non-specific antibody binding sites for 10 minutes. The cells were then stained with anti-HA monoclonal antibody (Abcam, USA, ab18181, 1:200 dilution) at 4°C overnight. Anti-mouse secondary antibody conjugated with Alexa Fluor 488 (CST, Danvers, MA, USA, 4408s, 1:1000 dilution) were added and incubated at room temperature in dark for 1 hour. DAPI was added to stain nucleus at room temperature in dark for 10 minutes. Fluorescence was detected using a Leica TCS SP5 II confocal fluorescence microscope (Leica, Germany).

### Western Blotting for Detection of *GHR* and Phosphorylated-STAT5

To assess the influence of *GHR* mutations on the expression of GHR protein, the GHR protein levels were determined by Western blotting method in HepG2 cells. HepG2 cells were transfected with GHR-WT or mutant GHR expression plasmids as described above. Total proteins were extracted using RIPA cell lysis buffer. 30 μg of proteins were separated by electrophoresis on 8% SDS-PAGE gels. The proteins were then transferred to nitrocellulose membranes (Applygen, Beijing, China) through a wet transfer method (Bio-Rad, California, USA) followed by the immunodetection. The primary rabbit anti-GHR antibody (Abcam, USA, ab65304, 1:1000 dilution) was incubated at 4°C overnight, followed by incubation with the HRP-coupled secondary antibody for 1 hour at room temperature (anti-rabbit antibody from CST, 7074, 1:3000 dilution). Signals were detected by using high sensitivity SuperSignal West Pico PLUS reagent (ThermoFisher Scientific, USA, 34577) and visualized using the Tanon 5200 Chemiluminescence imager (China). The bands were analyzed using Image J. A similar process was also conducted to assay β-actin as an internal reference.

To further investigate whether the mutant GHRs affect GH-induced signal transduction, the levels of the total and phosphorylated-STAT5 (p-STAT5) protein was further determined by Western blotting method in HepG2 cells. As described previously, HepG2 cells were transfected with GHR-WT or mutant GHR expression plasmids for 5 hours, and the cell culture medium was changed to low serum medium (1% fetal bovine serum) 16 hours. Then, 100 ng/ml rhGH was supplemented for 30 minutes. Total proteins were extracted with a phosphatase inhibitor cocktail (MedChemExpress, USA, HY-K0022) which protected the proteins from being dephosphorylated during protein extraction procedure. The following western blotting process were conducted as described above. The primary rabbit anti-phospho-STAT5-Y694 (Abcam, ab32364), rabbit anti-STAT5 (Abcam, ab16276) and rabbit anti-β-actin (CST, 4970s) were incubated at 1:1000 dilution at 4°C overnight, while the secondary anti-rabbit antibody (CST, 7074) was incubated at 1:2000 dilution for 1 hour at room temperature. All immunoblot data shown were representative of at least three independent experiments.

### Statistical Analysis

Each experiment was carried out three times. Samples in each group of experiments were repeated in triplicate. Normally distributed data are expressed as the mean ± standard deviation (SD), and skewness distribution data are expressed as the median. Statistical analysis was performed by t-test for two groups or one-way ANOVA for more than two groups. The Kruskal-Wallis test was used for skewed data. P<0.05 was considered significant. All statistical computations were performed with SPSS 23.0, and GraphPad Prism 6 was used for all statistical graphs.

## Results

### The Demographic Characteristics and Biochemical Measurements of LS Patients

The demographic characteristics and biochemical measurements of the four patients are shown in **Table 2**. All of the four patients with LS were male, the median age was 6.2 years old. Three patients had a severe short stature with height SDS of -5.49, -6.71 and -3.95, while the height SDS of patient 2 was -2.80. The median height was -4.72 SDS, and the median weight was -2.77 SDS.

**Table 2:** Clinical and biochemical features of patients with Laron Syndrome.

	Age	Gender	Birth weight (kg)	Birth length (cm)	Weight SDS	Height SDS	Basal GH (ng/ml)	Peak GH (ng/ml)	IGF-1 (ng/ml)	IGF-1 SDS	IGFBP3 (mg/L)	Bone age retardation (months)	GH treatment and duration	ΔHeight after rhGH (cm)
P1	7.6	Male	2.65	Unknown	-2.96	-5.49	15.44	23.36	32	-3.0	1.2 (1.3~5.6)	12	Yes, 0.053mg/kg/d for 2 months	3.3
P2	3.5	Male	3.15	46	-2.93	-2.80	2.10	Unknown	<25	-3.0	Unknown	6	No	
P3	14.3	Male	Unknown	Unknown	-2.61	-6.71	9.60	31.7	<25	-3.0	0.6 (0.7~3.9)	28	No	
P4	4.8	Male	3.20	51	-1.82	-3.95	1.59	>33.80	<25	-3.0	1.14 (1.0~4.7)	24	Yes, 0.057mg/kg/d for 32 months	17.8

SDS, standard deviation score; rhGH, recombinant human growth hormone.

As shown in **Table 2**, three patients (P1, P2, P3) had higher basal GH levels than the normal reference range (<2.0 ng/ml), ranging from 2.10 to 15.44 ng/ml, whereas the basal GH levels of P4 patient was 1.59 ng/ml (<2.0 ng/ml). The peak GH levels during the GH stimulation test were extremely high in P1, P3 and P4 patients with unknown peak GH levels of P2. However, IGF-1 levels of P2, P3 and P4 patients was less than the lower limit of normal range (<25 ng/ml), and IGF-1 concentration of P1 was only 32 ng/ml. As expected, all four patients had bone age retardation with the median retardation time of 18 months. Finally, none of the patients showed abnormalities in the pituitary MRI.

Patient 1 received recombinant human GH (rhGH) treatment at a dosage of 0.053 mg/kg/d for 2 months, and his height increased by 3.3 cm and IGF-1 increased from 32 ng/ml to 91 ng/ml. Patient 4 was given rhGH treatment at a dosage of 0.057 mg/kg/d for 32 months. During the last follow-up, the height increased by 17.8 cm (from -3.95 SDS to -2.96 SDS) with an annual growth rate of 6.8 cm/year, and IGF-1 increased from less than 25 ng/ml to 64 ng/ml.

### *GHR* Gene Variations of Four Patients With LS

**Figure 1:**
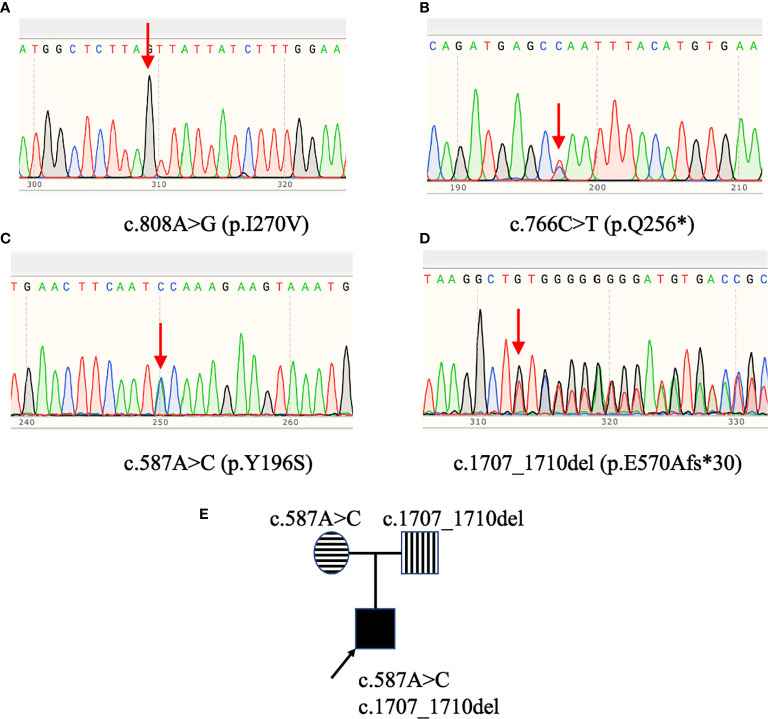
*GHR* gene variants in patients with Laron syndrome. Patient 2 **(A)** carried the c.808A>G (p.I270V) homozygous mutation. Patient 3 **(B)** harbored the c.766C>T (p.Q256*) heterozygous mutation. Patient 4 **(C, D)** had *GHR* gene compound heterozygous mutations: c.587A>C (p.Y196S) and c.1707_1710del (p.E570Afs*30). Pedigree analysis of patient 4 **(E)** revealed that the c.587A>C variant was inherited from his mother, while the c.1707_1710del variant was inherited from his father.

Exon 2 to exon 10 of *GHR* gene of four patients with LS were amplified and sequenced. The results were shown in **Table 3** and **Figure 1**. Two single nucleotide polymorphism (SNP) sites, c.1483C>A (p.P495T, rs6183) and c.1630A>C (p.I544L, rs6180), were detected in Patient 1. Patient 2 carried a novel homozygous mutation, c.808A>G (p.I270V) as shown in **Figure 1A**, and neither of his parents had the c.808A>G mutation. A novel heterozygous nonsense mutation, c.766C>T (p.Q256*) was identified in patient 3 as shown in **Figure 1B**, and the C>T base substitution led to a premature stop codon, which produced a truncated GHR protein. Patient 4 carried *GHR* gene compound heterozygous mutations including a novel heterozygous mutation, c.1707_1710del (p.E570Afs*30) in exon 10 (**Figure 1D**), which was inherited from his father and a previously reported heterozygous mutation, c.587A>C (p.Y196S, rs747888560) in exon 6 (**Figure 1C**), which was inherited from his mother as presented in **Figure 1E**. However, neither of his parents manifested a short phenotype. The height SDS of his father and mother was 0.14 SDS (177 cm) and -0.03 SDS (157 cm), respectively.

**Table 3:** Genetic variants of the *GHR* gene in patients with Laron Syndrome.

Patient	Exon	Nucleotide	Protein	rs number	Homozygote/Heterozygote	Novel	Clinical Significance
P1	Exon 10	c.1483C>A	p.P495T	6183	Heterozygote	No	Likely benign
	Exon 10	c.1630A>C	p.I544L	6180	Heterozygote	No	benign
P2	Exon 8	c.808 A>G	p.I270V		Homozygote	Yes	Uncertain significance
P3	Exon 7	c.766C>T	p.Q256*		Heterozygote	No	pathogenic
P4	Exon 6	c.587A>C	p.Y196S	747888560	Heterozygote	No	Likely pathogenic
	Exon 10	c.1707_1710del	p.E570Afs*30		Heterozygote	Yes	pathogenic

### Abnormal Subcellular Localization of Mutant *GHR*s Observed by Immunofluorescence Assays

**Figure 2:**
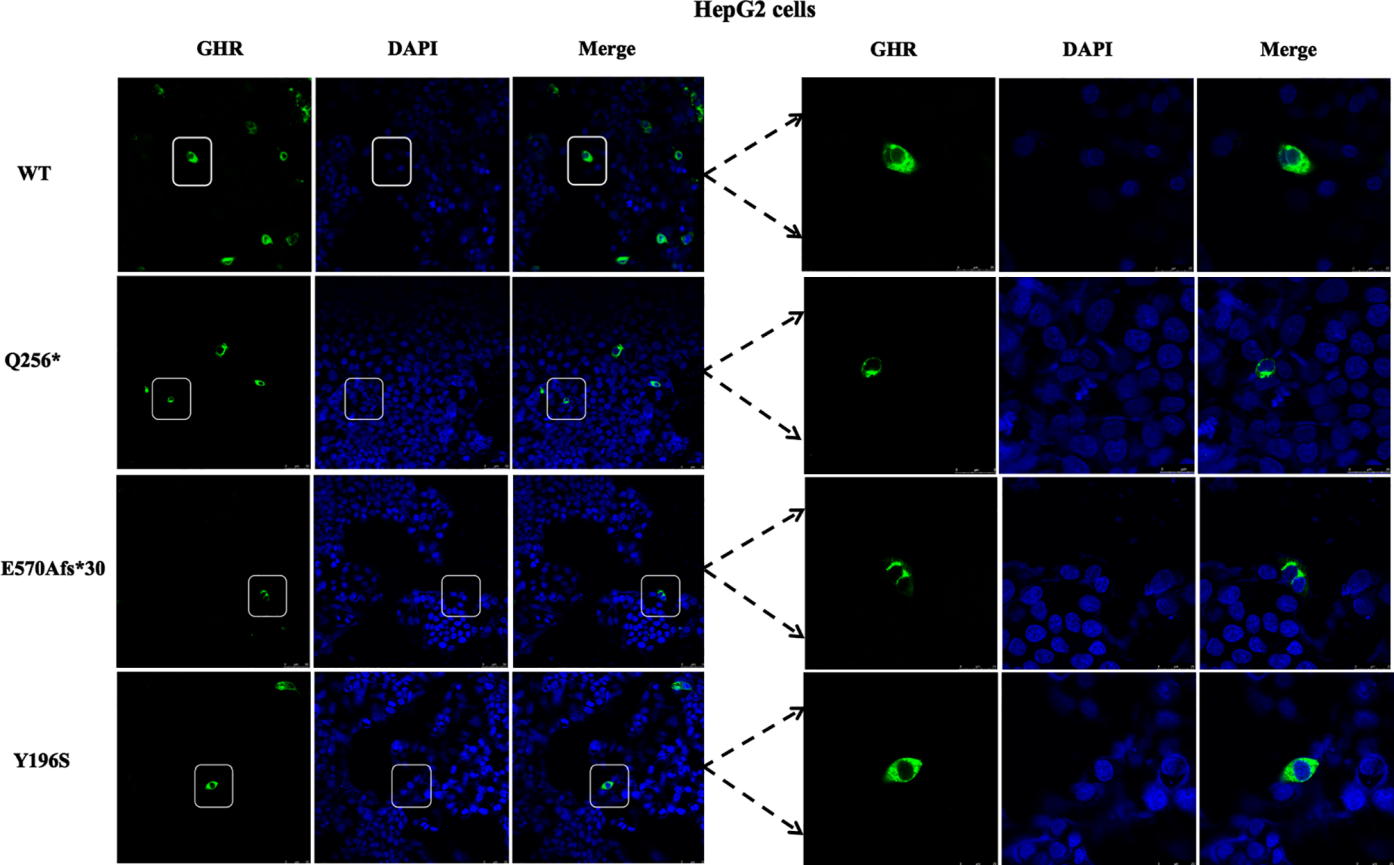
Immunofluorescence images of HepG2 cells transfected with *GHR* wild type (WT) and mutant expression plasmids (Q256*, E570Afs*30 and Y196S). The *GHR* protein was labeled by anti-HA primary antibody in green, the nucleus was labeled by DAPI in blue, and the merged images were displayed in the right column.

To visualize the mutant GHR subcellular distribution, GHR-WT and mutant GHR expression plasmids were transfected into HepG2 cells and immunofluorescence assay was performed with a monoclonal anti-HA tag (HA tag was inserted after the signal peptide in the extracellular domain) antibody. As presented in **Figure 2**, GHR-WT proteins in green color were evenly distributed on the cell membrane of HepG2 cells. However, when Q256* mutant GHR expression plasmids were transfected into HepG2 cells, mutant GHR proteins gathered around the nucleus and presented in a unique ring-like pattern, which was apparently different from the GHR-WT. Similar to the GHR-Q256* truncated protein, the same ring-like pattern was observed in cells transfected with GHR-E570Afs*30 expression plasmids, the mutant GHR proteins were concentrated in the cytoplasm. However, unlike the subcellular distribution of mutant GHR-Q256* and GHR-E570Afs*30, the mutant GHR-Y196S proteins had a similar subcellular localization of GHR-WT with an even GHR distribution on the cell membrane.

**Figure 3:**
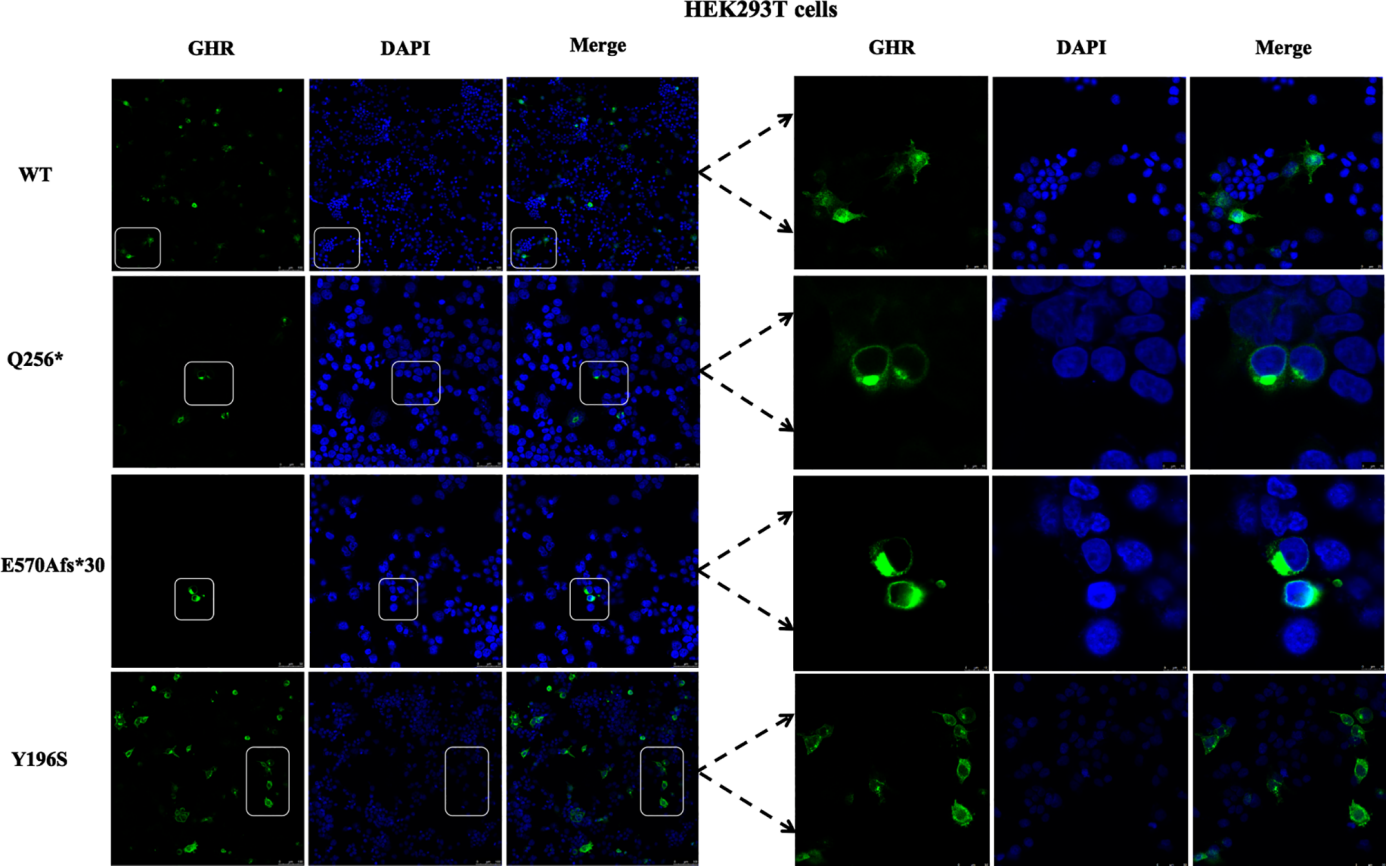
Immunofluorescence images of HEK293T cells transfected with *GHR* wild type (WT) and mutant expression plasmids (Q256*, E570Afs*30 and Y196S). The *GHR* protein was labeled by anti-HA primary antibody in green, the nucleus was labeled by DAPI in blue, and the merged images were displayed in the right column.

Next, the subcellular distribution of mutant GHRs were again conducted in HEK293T cells by the same transient transfection method and immunofluorescence assays as shown in **Figure 3**, and the localization of the GHR-WT and mutant GHRs were similar to that of HepG2 cells. GHR-WT proteins in HEK293T cells presented with a uniform distribution on cell surface as observed in HepG2 cells transfected with GHR-WT. Q256* truncated proteins and E570Afs*30 proteins in HEK293T cells were localized in a region adjacent to the nucleus, similar to the subcellular distribution pattern seen in HepG2 cells. In HEK293T cells transfected with Y196S, GHRs had a similar subcellular localization as GHR-WT, evenly distributed on the cell membrane.

### The Significantly Decreased Protein Levels of Mutant *GHR*s Transfected Into HepG2 Cells

**Figure 4:**
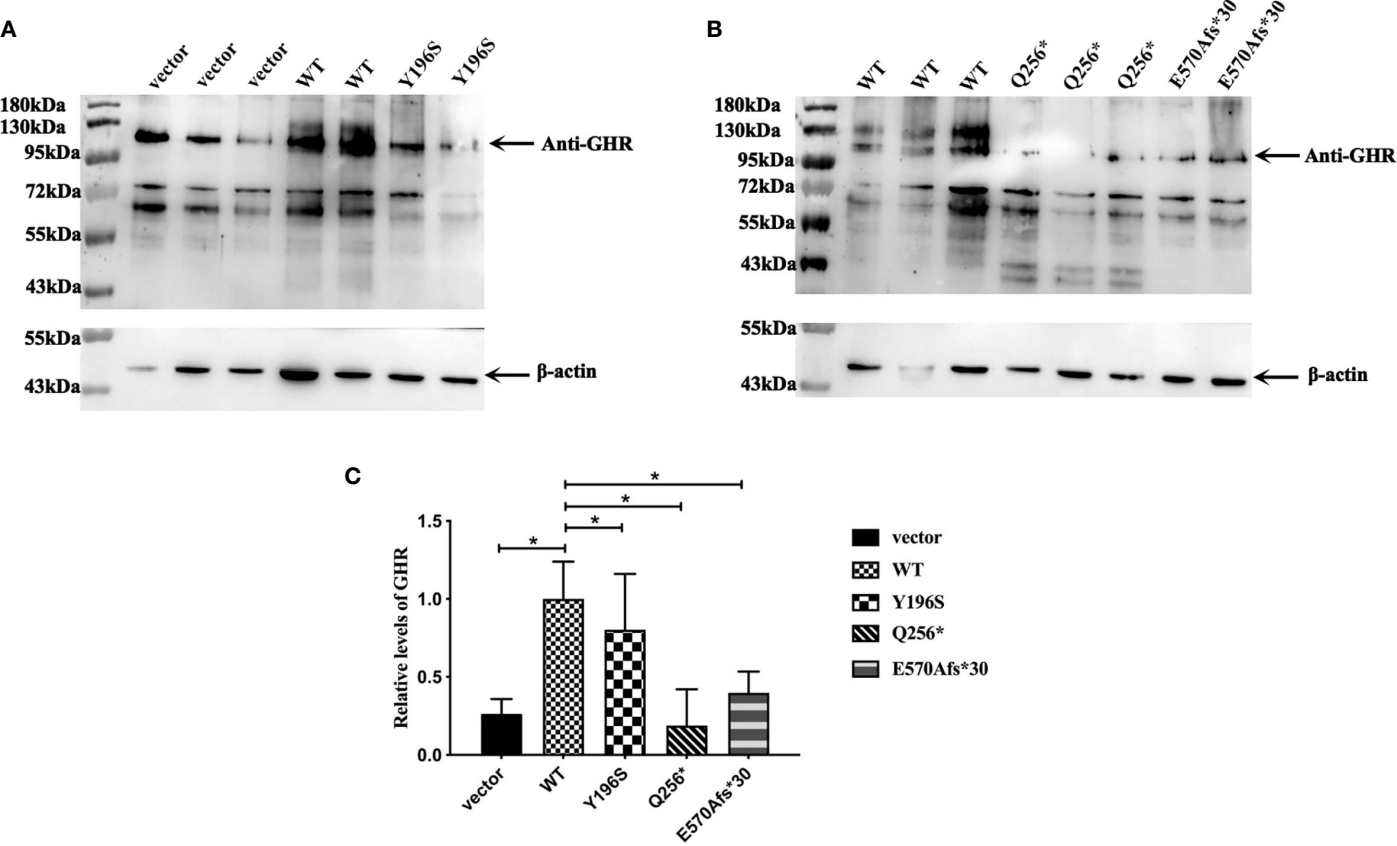
**(A, B)** Expression of *GHR* protein in HepG2 cells transfected with pcDNA3.1(+) empty vector, *GHR* wild type (WT) and mutant expression plasmids (Y196S, Q256* and E570Afs*30). The *GHR* protein has a molecular weight of around 130 kDa. Protein bands corresponding to 130 kDa could be detected in HepG2 cells transfected with Q256* and E570Afs*30, and the relative expression of both of them were significantly decreased compared to GHR-WT (**C**, P<0.05). HepG2 cells transfected with Q256* mutant plasmid produced a truncated protein, with a molecular with of around 43kD. However, no truncated protein was detected in HepG2 cells transfected with E570Afs*30 mutant plasmid.

Since the cellular localization of the mutant GHR has changed, has its protein levels also changed? In order to answer this issue, pcDNA3.1(+) empty vector, GHR-WT and mutant GHR expression plasmids were transfected into HepG2 cells, total proteins were extracted and Western blotting was performed. As shown in **Figures 4A, B**, GHRs could be detected in HepG2 cells transfected with pcDNA3.1(+) empty vector, and the molecular weight of GHRs were about 130 kDa. GHR-WT proteins were overexpressed compared to cells transfected with empty vector and the difference was statistically significant (**Figure 4C**, P<0.05). The GHR protein levels in HepG2 cells transfected with GHR-Y196S were significantly lower than that of GHR-WT and decreased by 19.65% as shown in **Figure 4C** (P<0.05). Specifically, unlike GHR-Y196S, HepG2 cells transfected with GHR-Q256* mutant expression plasmid produced a truncated protein, with a molecular weight of approximately 43 kDa as presented in **Figure 4B**. Protein bands corresponding to the molecular weight of 130kDa could be detected in HepG2 cells transfected with Q256*, and the GHR protein level was significantly decreased by 81.34% when compared to GHR-WT (P<0.05). Similar to GHR-Q256*, HepG2 cells transfected with GHR-E570Afs*30 mutant plasmid also had significantly diminished GHR protein expression at a molecular weight of 130kDa, which was decreased by 60.22% when compared to GHR-WT as presented in **Figure 4C** (P<0.05). However, no visible truncated GHR protein band was identified in western blot gel.

### The Significantly Decreased GH-Induced Phosphorylated STAT5 Levels of HepG2 Cells Transfected With Mutant *GHR*s

**Figure 5:**
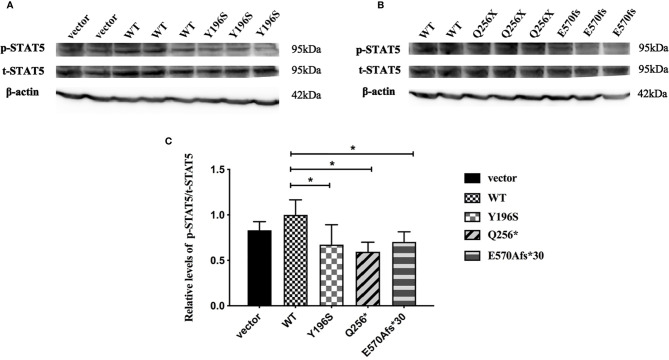
Phosphorylated STAT5 (p-STAT5) expression in HepG2 cells transfected with wild type *GHR* (WT), mutant *GHR* Y196S, Q256* and E570Afs*30 after 100ng/ml rhGH was added in the cell culture medium for 30 minutes. Both p-STAT5 and total STAT5 (t-STAT5) had a molecular weight of around 95 kDa **(A, B)**. The ratio of p-STAT5 to t-STAT5 was converted to gray-scale value by Image J. The expression of p-STAT5 was significantly decreased in *GHR*-Y196S, *GHR*-Q256* and *GHR*-E570Afs*30 when compared to *GHR*-WT as shown in **(C)** (P<0.05).

Since *GHR* gene mutations lead to abnormal subcellular location and the decreased expression of GHR proteins, it was not clear whether the transduction of post-receptor signal pathway was also affected. To further elucidate this problem, HepG2 cells were transfected with GHR-WT and mutant GHR expression plasmids, and cells were treated with 100ng/ml rhGH for 30 minutes before the total proteins were extracted. The levels of the total and p-STAT5 protein was measured by Western blotting. As shown in **Figures 5A, B**, both total STAT5 (t-STAT5) and p-STAT5 were detected at a molecular weight of 95 kDa in HepG2 cells. The relative levels of p-STAT5 among different groups was marked as the ratio of p-STAT5 to t-STAT5. As shown in **Figure 5C**, the levels of p-STAT5 proteins were significantly decreased in HepG2 cells transfected with GHR-Y196S mutant plasmid in comparison with cells transfected with GHR-WT, and decreased by 32.67% (P<0.05). In consistent with the results of GHR-Y196S, the levels of p-STAT5 proteins were also significantly reduced in HepG2 cells transfected with GHR-Q256* and GHR-E570Afs*30 mutant plasmids and reduced by 40.57% and 29.63%, respectively, as shown in **Figure 5C**, which indicated an impaired post-receptor signal transduction.

## Discussion

This study described the demographic characteristic and biochemical features of four Chinese patients with LS. Molecular genetic analysis revealed two novel *GHR* gene mutations, c.808A>G (p.I270V) and c.1707_1710del (p.E570Afs*30). Further *in vitro* functional experiments demonstrated that the novel *GHR* gene mutations disrupted the subcellular translocation of GHR proteins, affected the expression of GHR proteins and impaired the post-receptor signal transduction.

Patient 4 carried a compound heterozygous mutation, c.1707_1710del (p.E570Afs*30) and c.587A>C (p.Y196S). c.1707_1710del mutation was located in the intracellular region of GHR. Deletion of four bases resulted in a frameshift mutation, introducing a premature termination codon (TAG) at codon 600. The novel E570Afs*30 mutation influenced the subcellular distribution of GHR protein. Immunofluorescence demonstrated that the mutated GHR had a ring-like distribution pattern gathering around the nucleus, while GHR-WT were evenly distributed on the cell membrane. The GHR trafficking process from the endoplasmic reticulum to the cell membrane might be interrupted. E570Afs*30 mutation not only effected the translocation of GHR protein, but also influenced the expression of GHR and the GH-induced signal transduction. The expression of E570Afs*30 mutant protein was significantly decreased compared to the GHR-WT. The predicted consequence of this frameshift mutation would produce a truncated protein. However, no truncated protein was detected in Western blotting. The possible explanation of the result may be that the E570Afs*30 mutant *GHR* gene activated nonsense-mediated mRNA decay (30), which led to the GHR mRNA degradation. Milward et al. reported a homozygous 22-bp deletion in exon 10 of the GHR, resulting in a truncated GHR protein at amino acid 449 (GHR1-449) (31). Although both the GHR1-449 mutation and the E570Afs*30 mutation located in the intracellular domain, the mutant GHR1-449 protein had a similar cell surface distribution pattern as the GHR-WT, suggesting that GHR1-449 did not affect protein trafficking to the cell membrane, which was different from E570Afs*30 mutation in our study.

The other heterozygous mutation in patient 4, c.587A>C (p.Y196S), was first reported by Oh PS et al. in two Korean patients with LS. However, the *in vitro* cellular function studies were not performed in this study (32). The heterozygous c.587A>C substitution did not change the length of the GHR protein, and the mature Y196S mutant protein had the same molecular weight as the GHR-WT protein as demonstrated by Western blotting in our present study. However, the expression of GHR-Y196S was significantly lower than that of the GHR-WT, and the GHR post-receptor signal transduction was significantly impaired although the subcellular distribution of GHR-Y196S was similar to GHR-WT. Pedigree analysis revealed that the c.1707_1710del variant was inherited from his father, and the c.587A>C variant was inherited from his mother, nevertheless, neither of his parents had a short stature, suggesting that c.1707_1710del and the c.587A>C mutation had an additive effect on the phenotype of the patients.

In patient 3, a c.766C>T (p.Q256*) heterozygous nonsense mutation located in the extracellular domain was discovered. The substitution of C to T in exon 7 introduced a premature termination codon in place of a glutamic acid at amino acid 256 and produced a truncated GHR, leading to a deletion of the entire transmembrane domain and intracellular domain. The unique ring-like distribution pattern of GHR protein shown by immunofluorescence assay may be attributed to the mutant GHR-Q256* interfered with the translocation of mature GHR protein from the endoplasmic reticulum to the cell membrane. Western blot analysis demonstrated that the truncated protein had a molecular weight of 43 kDa, and the expression of both GHR and p-STAT5 were remarkably reduced compared to GHR-WT. The c.766C>T *GHR* gene mutation may explain the pathogenesis of the severe growth retardation in patient 3. A novel compound heterozygous mutation of the *GHR* gene, the c.724G>T (p.E224*) in exon 7 and c.981delC in exon 10, was reported by Kaji et al. in a Japanese girl whose height was -7.6 SDS (33). RT-PCR of the lymphocytes and sequencing of its cDNA revealed that no GHR mRNA was measured in the patient, suggesting that neither of the mutant alleles could generate a functional GHR mRNA.

In patient 2, a novel c.808A>G (p.I270V) homozygous missense mutation in the transmembrane domain was identified. *GHR* mutations occurring in the transmembrane domain were extremely rare. To our knowledge, in addition to c.839_875+1417del (D262Gfs*5) reported by Klammt et al. (27), c.875G>C (p.R274T) reported by Woods et al. (34) and c.800G>A (p.W267*) reported by Rughani et al. (25), the c.808A>G (p.I270V) in the present study was the only mutation located in the transmembrane domain of GHR. The I270V recombinant expression plasmid was not successfully constructed in our study. Therefore, the *in vitro* functional verification of this mutation was not performed in our present study.

**Figure 6:**
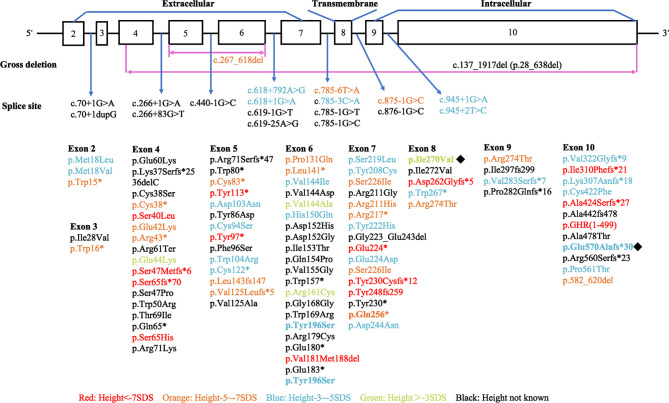
Diagrammatic representation of the human *GHR* gene mutations published in patients with Laron syndrome. Different colors stand for variant severity of height defect. Red stands for patients with height less than -7 SDS, orange stands for patients with height between -5 SDS to -7 SDS, blue stands for patients with height between -3 SDS to -5 SDS, and green stands for patients with height above -3 SDS. Novel mutations found in our present study were labeled with ◆.

Various mutations in the *GHR* gene have been documented, including missense, deletion, nonsense, frameshift, and splice site mutations, which affect the expression of GHR, ligand binding, GHR dimerization, or signal transduction and result in the dysfunction of GHR (4, 17). We performed a literature review to summarize the published *GHR* gene mutation locus as shown in **Figure 6** (detailed information could be found in **Supplementary Table 1**). Among the 120 known *GHR* mutations, 77 mutations occurred extracellularly, 17 occurred in the GHR introns, 17 occurred intracellularly, and 5 occurred in the transmembrane domain (detailed information was shown in **Supplementary Material**). The remaining four mutations were large fragments deletion. Mutations in transmembrane domain were extremely scarce. Storr et al. found that the expression of milder gene defects might show significant variability within each kindred, explaining why some patients were clinically easy to diagnose, whereas others may be under-diagnosed (5). The short stature phenotype did not always parallel the *GHR* gene defect. Among our four patients with LS, patient 3 had the most severe genetic defect, and the loss of function of one *GHR* allele (Q256*) led to a severe height deficit. However, as for patient 1, who manifested a severe short phenotype with a height of -5.49 SDS, no *GHR* gene mutation was detected. Further genetic study about this patient needs to be done in the future.

Recombinant human IGF-1 (rhIGF-1) replacement is the recommended treatment for severe LS (35). However, the efficacy in mild or moderate LS patients remains unclear. Moreover, due to the unavailability of rhIGF-1 in China, the management of patients with LS is difficult. A study of combined rhGH plus rhIGF-1 therapy in children with short stature, low IGF-1 (below -1.0 SD) and normal GH levels assessed growth responses according to the dose of rhIGF-1. In the group receiving the highest dose of rhIGF-1 (150 μg/kg/d) combined with rhGH at 45 μg/kg/d, the year 1 height velocity was 11.2 ± 2.1 compared with 9.3 ± 1.7 cm/y in the group receiving rhGH at 45 μg/kg/d alone (36). rhGH was administered in patient 4 at 57 μg/kg/d for 32 months in our center, and the growth velocity was 6.8 cm/y with the height increased by 17.8 cm in total. The IGF-1 level increased from 25 ng/ml to 64 ng/ml. This finding may be attributed to the direct effect of GH to promote epiphyseal growth. Our clinical experience provides evidence and possibility in growth-promotion with rhGH under circumstance of unavailability of rhIGF-1.

Several limitations existed in our study. Since the phenotype of the patients was typical of LS, we conducted Sanger sequencing of the *GHR* gene, it was unclear whether defects of post-receptor components exist in the GH signal transduction pathway, such as *STAT5B, IGFALS, IGF-1* and *PAPPA2* genes. Besides, overlapping phenotypes and attenuated presentations can complicate the clinical picture, in which whole-exon sequencing or even whole-genome sequencing should be performed to discover underlying genetic abnormalities.

## Data Availability Statement

The datasets presented in this study can be found in online repositories. The names of the repository/repositories and accession number(s) can be found below: GenBank [accession: MW701347-MW701349].

## Ethics Statement

The studies involving human participants were reviewed and approved by Ethics Committee of Peking Union Medical College Hospital. Written informed consent to participate in this study was provided by the participants’ legal guardian/next of kin. Written informed consent was obtained from the individual(s), and minor(s)’ legal guardian/next of kin, for the publication of any potentially identifiable images or data included in this article.

## Author Contributions

RL conducted the *in vitro* experiments and finished the rough draft. FG and HZ designed the experiments and revised the draft, and FG polished the language. HP supervised the experiments. HL and HM helped with the genetic data analysis. YZ and SC contribute to statistical analysis. LD, HY and LW revised the initial draft and provided useful suggestions. All authors agree to be accountable for the content of the work. All authors contributed to the article and approved the submitted version.

## Funding

The study was supported by grants of The National Key Research and Development Program of China (No.2016YFC0901501) and the CAMS Innovation Fund for Medical Sciences (CAMS-2016-I2M-1-002).

## Conflict of Interest

The authors declare that the research was conducted in the absence of any commercial or financial relationships that could be construed as a potential conflict of interest.

## Abbreviations

LS, Laron syndrome; GHR, Growth hormone receptor; GHR-WT, GHR wild type; rhGH, Recombinant human growth hormone; rhIGF-1, Recombinant human IGF-1.
