# Supplementary material for: Identification and In Vitro Functional Verification of Two Novel Mutations of GHR Gene in the Chinese Children with Laron Syndrome
Source: Front Endocrinol (Lausanne). 2021 Apr 12;12:605736. doi: 10.3389/fendo.2021.605736 (PMC8072467; doi:10.3389/fendo.2021.605736)
Supplement: Supplementary file 1 [file Table_1.docx]

**Supplementary Table 1.** Published human *GHR* gene mutations in patients with Laron syndrome.

| **Exon** | **Nucleotide change** | **Protein change** | **Mutation Type** | **Height SDS** | **Reference** |
| --- | --- | --- | --- | --- | --- |
| 2 | c.1A>T | p.Met18Leu | Missense | -3.76 | [1] |
| 2 | c.1A>G | p.Met18Val | Missense | -3.76 | [1] |
| 2 | c.11G>A | p.Trp15X | Nonsense | -5.6/-6.8/-4.7 | [2] |
| 2 | c.12G>A | p.Trp15X | Nonsense | Not known | [3] |
| Intron2 | c.70+1G>A | (exon 2 skipping) | Splice site | Not known | [4] |
| Intron2 | c.70+1dupG | p.Ala6GlyfsX7 | Splice site | Not known | [5] |
| 3 | c.82A>G | p.Ile28Val | Missense | Not known |  |
| 3 | c.101G>A | p.Trp16X | Nonsense | Not known | [3] |
| 3 | c.102G>A | p.Trp16X | Nonsense | -5.5 | [6] |
| 4 | c.148G>A | p.Glu60Lys | Missense | Not known |  |
| 4 | c.161-162delC | p.Lys37SerfsX25 | Small deletion | Not known | [4] |
| 4 | c.162delC | 36delC | Frameshift | Not known | [4] |
| 4 | c.166T>A | p.Cys38Ser | Missense | Not known | [4] |
| 4 | c.168C>A | p.Cys38X | Nonsense | -5.5 | [6, 7] |
| 4 | c.173C>T | p.Ser40Leu | Missense | -7.90 | [8] |
| 4 | c.178G>A | p.Glu42Lys | Missense | -5.92 | [9] |
| 4 | c.181C>T | p.Arg43X | Nonsense | -3.9/-7.0 | [7, 10, 11] |
| 4 | c.182G>A | p.Arg61Ter | Missense | Not known |  |
| 4 | c.184G>A | p.Glu44Lys | Missense | -2.9 | [12] |
| 4 | c.189-193delTT | p.Ser47MetfsX6 | Small deletion | -8.4/-8.5 | [11] |
| 4 | c.192-193delTT | p.Ser65fsX70  46delTT | Frameshift | -8.5 | [11] |
| 4 | c.193T>C | p.Ser47Pro | Missense | Not known |  |
| 4 | c.202T>C | p.Trp50Arg | Missense | Not known | [4] |
| 4 | c.206C>T | p.Thr69Ile | Missense | Not known |  |
| 4 | c.247C>T | p.Gln65X | Nonsense | Not known | [4] |
| 4 | c.249T>C | p.Ser65His | Missense | -6.8/-8.2 | [13] |
| 4 | c.266G>A | p.Arg71Lys | Missense | Not known | [14] |
| Intron4 | c.266+1G>A | p.Asn28ArgfsX41  (exon2 skipping) | Splice site | Not known | [14] |
| 5 | c.267_439del | p.Arg71SerfsX47  (exon5 skipping) | Gross deletion | Not known | [15] |
| 5 | c.293G>A | p.Trp80X | Nonsense | Not known | [4] |
| 5 | c.303C>A | p.Cys83X | Nonsense | -5.28 | [16] |
| 5 | c.338dupA | p.Tyr113X | Nonsense | -8.0 | [17] |
| 5 | c.307G>A | p.Asp103Asn | Missense | -3.76 | [1] |
| 5 | c.310T>G | p.Tyr86Asp | Missense | Not known | [18] |
| 5 | c.335G>C | p.Cys94Ser | Missense | -4.8/-5.0 | [19] |
| 5 | c.338dupA | p.Tyr97X | Nonsense | -8.0 | [17] |
| 5 | c.341T>C | p.Phe96Ser | Missense | Not known | [20] |
| 5 | c.346C>A | p.Cys83X | Nonsense | -5.28 | [16] |
| 5 | c.364T>G | p.Trp104Arg | Missense | -3.76 | [8] |
| 5 | c.420T>A | p.Cys122X | Nonsense | -3.2 | [12] |
| 5 | c.421_422dupTT | p.Leu143fs147  422insTT | Frameshift | -5.0 | [21] |
| 5 | c.422insTT | p.Val125LeufsX5 | Small insertion | -5.0 | [21] |
| 5 | c.428T>C | p.Val125Ala | Missense | Not known | [14] |
| Intron5 | c.440-1G>C | p.Val129AspfsX18  (exon6 skipping) | Splice site | Not known | [14] |
| 6 | c.446C>A | p.Pro131Gln | Missense | -6.3 | [22] |
| 6 | c.476T>A | p.Leu141X | Nonsense | -5.6/-6.8/-4.7 | [2] |
| 6 | c.484G>A | p.Val144Ile | Missense | -3.09 | [23] |
| 6 | c.485T>A | p.Val144Asp | Missense | Not known | [14] |
| 6 | c.485T>C | p.Val144Ala | Missense | -2.65 | [18, 24] |
| 6 | c.504T>G | p.His150Gln | Missense | -4.8/-5.0 | [19] |
| 6 | c.508G>C | p.Asp152His | Missense | Not known | [25] |
| 6 | c.509A>G | p.Asp152Gly | Missense | Not known | [26] |
| 6 | c.512T>C | p.Ile153Thr | Missense | Not known | [27] |
| 6 | c.515A>C | p.Gln154Pro | Missense | Not known | [27] |
| 6 | c.518T>G | p.Val155Gly | Missense | Not known | [27] |
| 6 | c.524G>A | p.Trp157X | Nonsense | Not known | [4] |
| 6 | c.525G>A | p.Trp157X | Nonsense | Not known | [4] |
| 6 | c.535C>T | p.Arg161Cys | Missense | -2.9 | [12, 14] |
| 6 | c.558A>G | p.Gly168Gly | Synonymous | Not known | [5] |
| 6 | c.559T>C | p.Trp169Arg | Missense | Not known | [28] |
| 6 | c.587A>C | p.Tyr196Ser | Missense | -3.95 | [29] |
| 6 | c.591C>T | p.Arg179Cys | Missense | Not known | [30] |
| 6 | c.592G>T | p.Glu180X | Nonsense | Not known |  |
| 6 | c.594A>G | p.Val181_Met188del  (aka E180 splice) | Splice site | -6.7~-10.0 | [31] |
| 6 | c.601G>T | p.Glu183X | Nonsense | Not known | [32] |
| Intron6 | c.618+792A>G | p.Met188_Met189ins36 (E180 splice) | Splice site | -3.3/-5.6 | [33] |
| Intron6 | c.619-1G>T | p.Met189IlefsX8  (exon7 skipping) | Splice site | Not known | [11] |
| 7 | c.656C>T | p.Ser219Leu | Missense | -3.85 | [34] |
| 7 | c.677A>G | p.Tyr208Cys | Missense | -4.5/-4.0 | [35] |
| 7 | c.679G>T | p.Ser226Ile | Missense | -6.7 | [36] |
| 7 | c.685C>G | p.Arg211Gly | Missense | Not known | [14] |
| 7 | c.686G>A | p.Arg211His | Missense | -5.1 | [12] |
| 7 | c.703C>T | p.Arg217X | Nonsense | -4.4/-8.9/-5.4/-5.8 | [11, 37] |
| 7 | c.718T>C | p.Tyr222His | Missense | -3.0 | [38] |
| 7 | c.723C>T | p.Gly223_Glu243del  or p.Gly223Gly | Splice site | Not known | [4] |
| 7 | c.724G>T | p.Glu224X | Nonsense | -7.6 | [39] |
| 7 | c.726G>C | p.Glu224Asp | Missense | -3.2 | [12] |
| 7 | c.731G>T | p.Ser226Ile | Missense | -6.7 | [36] |
| 7 | c.742-744del2 | p.Tyr230CysfsX12 | Small deletion | -8.1 | [11] |
| 7 | c.743-744delAT | p.Tyr248fs259  230delAT | Nonsense | -8.1 | [11] |
| 7 | c.744delT | p.Tyr230X | Small deletion | Not known | [18] |
| **7** | **c.766C>T** | **p.Gln256X** | **Nonsense** | **-6.71** | **Present Study** |
| 7 | c.784G>A | p.Asp244Asn | Missense | -4.5/-4.0 | [35] |
| Intron7 | c.785-6T>A | p.Asp244GlyfsX5  (exon8 skipping) | Splice site | -6.0 | [40] |
| Intron7 | c.785-3C>A | p.Asp244GlyfsX5  (exon8 skipping) | Splice site | -3.05 | [41] |
| Intron7 | c.785-1G>T | p.Asp244GlyfsX5  (exon8 skipping) | Splice site | Not known | [18] |
| Intron7 | c.785-1G>C | p.Asp244GlyfsX5  (exon8 skipping) | Splice site | Not known |  |
| 8 | c.800G>A | p.Trp267* | Nonsense | -3.5 | [42] |
| **8** | **c.808A>G** | **p.Ile270Val** | **Missense** | **-2.80** | **Present study** |
| 8 | c.875G>C | p.Arg274Thr | Splice site | -5.4/-5.2 | **[43]** |
| 8 | c.839-875+1417del | p.Asp262GlyfsX5 | deletion | -9.11 | [44] |
| Intron8 | c.875-1G>C | p.Asp244GlyfsX5  (exon8 skipping) | Splice site | -5.4/-5.2 | [43] |
| Intron8 | c.876-1G>C | p.Ile275LysfsX4  (exon9 skipping) | Splice site | Not known | [45] |
| 9 | c.889-911del | p.Ile297fs299 | Small deletion | Not knwon |  |
| 9 | c.895-899dupC | p.Val283SerfsX7 | Small insertion | -4.0 | [46] |
| 9 | c.899dupC |  | Dominant negative effect | -4.07 | [47] |
| 9 | c.899-911del13 | p.Pro282GlnfsX16 | Small deletion | Not known | [15] |
| Intron9 | c.945+1G>A | p.Ile275LysfsX4  (exon9 skipping) | Splice site | -3.0/-3.5 | [48] |
| Intron9 | c.945+2T>C | Lost Box 1 | Frameshift | -4.3 | [49] |
| 9 | Removal of exon9 | Truncated GHR 1-279 | Frameshift | Not known | [3, 50] |
| 10 | c.964dupG | p.Val322GlyfsX9 | Insertion | -3.09 | [49] |
| 10 | c.981delC | p.Ile310PhefsX21  or p.Ile310fs | Frameshift | -7.6 | [39] |
| 10 | c.920_921insTCTCAAAGATTACA | p.Lys307AsnfsX18 | insertion | -3.1 | [3, 49] |
| 10 | c.1319G>T | p.Cys422Phe | Missense | -4.8 | [51] |
| 10 | c.1323-1344del22 | p.Ala424SerfsX27 | Small deletion | -8.7 | [52] |
| 10 | c.1324-1345del | p.Ala442fs478 | Small deletion | Not known |  |
| 10 | c.1342_1345del | p.GHR(1-499) | Small deletion | -8.7/-6.0 | [52] |
| 10 | c.1486G>A | p.Ala478Thr | Missense | Not known | [53] |
| **10** | **c.1707-1710del** | **p.Glu570fs** | **Frameshift** | **-3.95** | **Present study** |
| 10 | c.1733-1734delG | p.Arg560SerfsX23 | Small deletion | Not known | [29] |
| 10 | c.1735C>A | p.Pro561Thr | Missense | -4.8 | [51] |
| 10 | c.1776delG | p.582_620del | Small deletion | -5.28 | [16] |
| 3,5,6 | c.71_136del,  267_618del | p.Ala6_Asn28delinsAsp,  Asn72TrpfsX5  (exons3,5,6 missing) | Gross deletions | -4.2/-7.2/-8.7 | [54] |
| 5,6 | c.267_618del | Asn72TrpfsX5  (exons5,6 missing) | Gross deletions | -4.2/-7.3 | [55] |
| 4-10 | c.137_1917del | p.28_638del | Gross deletions | Not known | [56] |
| 4-10 | Del(5)p11-p13.1 | del exon4-10 | Nonsense | Not known | [17] |

**References**

1. Moia S, Tessaris D, Einaudi S, de Sanctis L, Bona G, Bellone S, et al. Compound heterozygosity for two GHR missense mutations in a patient affected by Laron Syndrome: a case report. Italian journal of pediatrics. 2017;43(1):94.

2. Shevah O, Borrelli P, Rubinstein M, Laron Z. Identification of two novel mutations in the human growth hormone receptor gene. J Endocrinol Invest. 2003;26(7):604-8.

3. Lin S, Li C, Li C, Zhang X. Growth Hormone Receptor Mutations Related to Individual Dwarfism. International journal of molecular sciences. 2018;19(5).

4. Sobrier ML, Dastot F, Duquesnoy P, Kandemir N, Yordam N, Goossens M, et al. Nine novel growth hormone receptor gene mutations in patients with Laron syndrome. The Journal of clinical endocrinology and metabolism. 1997;82(2):435-7.

5. Arman A, Ozon A, Isguven PS, Coker A, Peker I, Yordam N. Novel splice site mutation in the growth hormone receptor gene in Turkish patients with Laron-type dwarfism. J Pediatr Endocrinol Metab. 2008;21(1):47-58.

6. Pantel J, Grulich-Henn J, Bettendorf M, Strasburger CJ, Heinrich U, Amselem S. Heterozygous nonsense mutation in exon 3 of the growth hormone receptor (GHR) in severe GH insensitivity (Laron syndrome) and the issue of the origin and function of the GHRd3 isoform. The Journal of clinical endocrinology and metabolism. 2003;88(4):1705-10.

7. Amselem S, Sobrier ML, Duquesnoy P, Rappaport R, Postel-Vinay MC, Gourmelen M, et al. Recurrent nonsense mutations in the growth hormone receptor from patients with Laron dwarfism. The Journal of clinical investigation. 1991;87(3):1098-102.

8. Arman A, Yüksel B, Coker A, Sarioz O, Temiz F, Topaloglu AK. Novel growth hormone receptor gene mutation in a patient with Laron syndrome. J Pediatr Endocrinol Metab. 2010;23(4):407-14.

9. Chen X, Song F, Dai Y, Bao X, Jin Y. A novel mutation of the growth hormone receptor gene (GHR) in a Chinese girl with Laron syndrome. J Pediatr Endocrinol Metab. 2003;16(8):1183-9.

10. Putzolu M, Meloni A, Loche S, Pischedda C, Cao A, Moi P. A homozygous nonsense mutation of the human growth hormone receptor gene in a Sardinian boy with Laron-type dwarfism. J Endocrinol Invest. 1997;20(5):286-8.

11. Berg MA, Argente J, Chernausek S, Gracia R, Guevara-Aguirre J, Hopp M, et al. Diverse growth hormone receptor gene mutations in Laron syndrome. American journal of human genetics. 1993;52(5):998-1005.

12. Goddard AD, Covello R, Luoh SM, Clackson T, Attie KM, Gesundheit N, et al. Mutations of the growth hormone receptor in children with idiopathic short stature. The Growth Hormone Insensitivity Study Group. The New England journal of medicine. 1995;333(17):1093-8.

13. Ying YQ, Wei H, Cao LZ, Lu JJ, Luo XP. Clinical features and growth hormone receptor gene mutations of patients with Laron syndrome from a Chinese family. Zhongguo dang dai er ke za zhi = Chinese journal of contemporary pediatrics. 2007;9(4):335-8.

14. Amselem S, Duquesnoy P, Duriez B, Dastot F, Sobrier ML, Valleix S, et al. Spectrum of growth hormone receptor mutations and associated haplotypes in Laron syndrome. Human molecular genetics. 1993;2(4):355-9.

15. Gastier JM, Berg MA, Vesterhus P, Reiter EO, Francke U. Diverse deletions in the growth hormone receptor gene cause growth hormone insensitivity syndrome. Human mutation. 2000;16(4):323-33.

16. Tiulpakov A, Rubtsov P, Dedov I, Peterkova V, Bezlepkina O, Chrousos GP, et al. A novel C-terminal growth hormone receptor (GHR) mutation results in impaired GHR-STAT5 but normal STAT-3 signaling. The Journal of clinical endocrinology and metabolism. 2005;90(1):542-7.

17. Diniz ET, Jorge AA, Arnhold IJ, Rosenbloom AL, Bandeira F. Novel nonsense mutation (p.Y113X) in the human growth hormone receptor gene in a Brazilian patient with Laron syndrome. Arq Bras Endocrinol Metabol. 2008;52(8):1264-71.

18. Shevah O, Rubinstein M, Laron Z. Molecular defects of the growth hormone receptor gene, including a new mutation, in Laron syndrome patients in Israel: relationship between defects and ethnic groups. The Israel Medical Association journal : IMAJ. 2004;6(10):630-3.

19. Fang P, Riedl S, Amselem S, Pratt KL, Little BM, Haeusler G, et al. Primary growth hormone (GH) insensitivity and insulin-like growth factor deficiency caused by novel compound heterozygous mutations of the GH receptor gene: genetic and functional studies of simple and compound heterozygous states. The Journal of clinical endocrinology and metabolism. 2007;92(6):2223-31.

20. Rosenbloom AL, Guevara-Aguirre J. Lessons from the genetics of laron syndrome. Trends in endocrinology and metabolism: TEM. 1998;9(7):276-83.

21. Gennero I, Edouard T, Rashad M, Bieth E, Conte-Aurio F, Marin F, et al. Identification of a novel mutation in the human growth hormone receptor gene (GHR) in a patient with Laron syndrome. J Pediatr Endocrinol Metab. 2007;20(7):825-31.

22. Walker JL, Crock PA, Behncken SN, Rowlinson SW, Nicholson LM, Boulton TJ, et al. A novel mutation affecting the interdomain link region of the growth hormone receptor in a Vietnamese girl, and response to long-term treatment with recombinant human insulin-like growth factor-I and luteinizing hormone-releasing hormone analogue. The Journal of clinical endocrinology and metabolism. 1998;83(7):2554-61.

23. Pagani S, Petkovic V, Messini B, Meazza C, Bozzola E, Mullis PE, et al. Heterozygous GHR gene mutation in a child with idiopathic short stature. J Pediatr Endocrinol Metab. 2014;27(3-4):329-34.

24. Bonioli E, Tarò M, Rosa CL, Citana A, Bertorelli R, Morcaldi G, et al. Heterozygous mutations of growth hormone receptor gene in children with idiopathic short stature. Growth hormone & IGF research : official journal of the Growth Hormone Research Society and the International IGF Research Society. 2005;15(6):405-10.

25. Duquesnoy P, Sobrier ML, Duriez B, Dastot F, Buchanan CR, Savage MO, et al. A single amino acid substitution in the exoplasmic domain of the human growth hormone (GH) receptor confers familial GH resistance (Laron syndrome) with positive GH-binding activity by abolishing receptor homodimerization. The EMBO journal. 1994;13(6):1386-95.

26. Yang C, Chen JY, Lai CC, Lin HC, Yeh GC, Hsu HH. Clinical, biochemical and molecular investigations of three Taiwanese children with Laron syndrome. J Pediatr Endocrinol Metab. 2004;17(2):165-71.

27. Wojcik J, Berg MA, Esposito N, Geffner ME, Sakati N, Reiter EO, et al. Four contiguous amino acid substitutions, identified in patients with Laron syndrome, differently affect the binding affinity and intracellular trafficking of the growth hormone receptor. The Journal of clinical endocrinology and metabolism. 1998;83(12):4481-9.

28. Porto WF, Marques FA, Pogue HB, de Oliveira Cardoso MT, do Vale MGR, da Silva Pires Á, et al. Computational Investigation of Growth Hormone Receptor Trp169Arg Heterozygous Mutation in a Child With Short Stature. Journal of cellular biochemistry. 2017;118(12):4762-71.

29. David A, Hwa V, Metherell LA, Netchine I, Camacho-Hubner C, Clark AJ, et al. Evidence for a continuum of genetic, phenotypic, and biochemical abnormalities in children with growth hormone insensitivity. Endocr Rev. 2011;32(4):472-97.

30. Meyer S, Ipek M, Keth A, Minnemann T, von Mach MA, Weise A, et al. Short stature and decreased insulin-like growth factor I (IGF-I)/growth hormone (GH)-ratio in an adult GH-deficient patient pointing to additional partial GH insensitivity due to a R179C mutation of the growth hormone receptor. Growth hormone & IGF research : official journal of the Growth Hormone Research Society and the International IGF Research Society. 2007;17(4):307-14.

31. Jorge AA, Menezes Filho HC, Lins TS, Guedes DR, Damiani D, Setian N, et al. [Founder effect of E180splice mutation in growth hormone receptor gene (GHR) identified in Brazilian patients with GH insensitivity]. Arq Bras Endocrinol Metabol. 2005;49(3):384-9.

32. Berg MA, Peoples R, Pérez-Jurado L, Guevara-Aguirre J, Rosenbloom AL, Laron Z, et al. Receptor mutations and haplotypes in growth hormone receptor deficiency: a global survey and identification of the Ecuadorean E180splice mutation in an oriental Jewish patient. Acta paediatrica (Oslo, Norway : 1992) Supplement. 1994;399:112-4.

33. Metherell LA, Akker SA, Munroe PB, Rose SJ, Caulfield M, Savage MO, et al. Pseudoexon activation as a novel mechanism for disease resulting in atypical growth-hormone insensitivity. American journal of human genetics. 2001;69(3):641-6.

34. Hattori A, Katoh-Fukui Y, Nakamura A, Matsubara K, Kamimaki T, Tanaka H, et al. Next generation sequencing-based mutation screening of 86 patients with idiopathic short stature. Endocrine journal. 2017;64(10):947-54.

35. Enberg B, Luthman H, Segnestam K, Ritzén EM, Sundström M, Norstedt G. Characterisation of novel missense mutations in the GH receptor gene causing severe growth retardation. European journal of endocrinology. 2000;143(1):71-6.

36. Jorge AA, Souza SC, Arnhold IJ, Mendonca BB. The first homozygous mutation (S226I) in the highly-conserved WSXWS-like motif of the GH receptor causing Laron syndrome: supression of GH secretion by GnRH analogue therapy not restored by dihydrotestosterone administration. Clinical endocrinology. 2004;60(1):36-40.

37. Fassone L, Corneli G, Bellone S, Camacho-Hübner C, Aimaretti G, Cappa M, et al. Growth hormone receptor gene mutations in two Italian patients with Laron Syndrome. J Endocrinol Invest. 2007;30(5):417-20.

38. Tauber MT, Porra V, Dastot F, Molinas C, Amselem S, Cholin S, et al. Heterozygous mutation in the WSXWS equivalaent motif of the growth hormone receptor in a child with poor response to growth hormone therapy. Growth hormone & IGF research : official journal of the Growth Hormone Research Society and the International IGF Research Society. 1998;8(3):211-6.

39. Kaji H, Nose O, Tajiri H, Takahashi Y, Iida K, Takahashi T, et al. Novel compound heterozygous mutations of growth hormone (GH) receptor gene in a patient with GH insensitivity syndrome. The Journal of clinical endocrinology and metabolism. 1997;82(11):3705-9.

40. David A, Miraki-Moud F, Shaw NJ, Savage MO, Clark AJ, Metherell LA. Identification and characterisation of a novel GHR defect disrupting the polypyrimidine tract and resulting in GH insensitivity. European journal of endocrinology. 2010;162(1):37-42.

41. Aalbers AM, Chin D, Pratt KL, Little BM, Frank SJ, Hwa V, et al. Extreme elevation of serum growth hormone-binding protein concentrations resulting from a novel heterozygous splice site mutation of the growth hormone receptor gene. Hormone research. 2009;71(5):276-84.

42. Rughani A, Zhang D, Vairamani K, Dauber A, Hwa V, Krishnan S. Severe growth failure associated with a novel heterozygous nonsense mutation in the GHR transmembrane domain leading to elevated growth hormone binding protein. Clinical endocrinology. 2020;92(4):331-7.

43. Woods KA, Fraser NC, Postel-Vinay MC, Savage MO, Clark AJ. A homozygous splice site mutation affecting the intracellular domain of the growth hormone (GH) receptor resulting in Laron syndrome with elevated GH-binding protein. The Journal of clinical endocrinology and metabolism. 1996;81(5):1686-90.

44. Klammt J, Shen S, Kiess W, Kratzsch J, Stobbe H, Vogel M, et al. Clinical and biochemical consequences of an intragenic growth hormone receptor (GHR) deletion in a large Chinese pedigree. Clinical endocrinology. 2015;82(3):453-61.

45. Ayling RM, Ross R, Towner P, Von Laue S, Finidori J, Moutoussamy S, et al. A dominant-negative mutation of the growth hormone receptor causes familial short stature. Nature genetics. 1997;16(1):13-4.

46. Aisenberg J, Auyeung V, Pedro HF, Sugalski R, Chartoff A, Rothenberg R, et al. Atypical GH insensitivity syndrome and severe insulin-like growth factor-I deficiency resulting from compound heterozygous mutations of the GH receptor, including a novel frameshift mutation affecting the intracellular domain. Hormone research in paediatrics. 2010;74(6):406-11.

47. Derr MA, Aisenberg J, Fang P, Tenenbaum-Rakover Y, Rosenfeld RG, Hwa V. The growth hormone receptor (GHR) c.899dupC mutation functions as a dominant negative: insights into the pathophysiology of intracellular GHR defects. The Journal of clinical endocrinology and metabolism. 2011;96(11):E1896-904.

48. Iida K, Takahashi Y, Kaji H, Nose O, Okimura Y, Abe H, et al. Growth hormone (GH) insensitivity syndrome with high serum GH-binding protein levels caused by a heterozygous splice site mutation of the GH receptor gene producing a lack of intracellular domain. The Journal of clinical endocrinology and metabolism. 1998;83(2):531-7.

49. Vairamani K, Merjaneh L, Casano-Sancho P, Sanli ME, David A, Metherell LA, et al. Novel Dominant-Negative GH Receptor Mutations Expands the Spectrum of GHI and IGF-I Deficiency. Journal of the Endocrine Society. 2017;1(4):345-58.

50. Soendergaard C, Young JA, Kopchick JJ. Growth Hormone Resistance-Special Focus on Inflammatory Bowel Disease. International journal of molecular sciences. 2017;18(5).

51. Kou K, Lajara R, Rotwein P. Amino acid substitutions in the intracellular part of the growth hormone receptor in a patient with the Laron syndrome. The Journal of clinical endocrinology and metabolism. 1993;76(1):54-9.

52. Milward A, Metherell L, Maamra M, Barahona MJ, Wilkinson IR, Camacho-Hübner C, et al. Growth hormone (GH) insensitivity syndrome due to a GH receptor truncated after Box1, resulting in isolated failure of STAT 5 signal transduction. The Journal of clinical endocrinology and metabolism. 2004;89(3):1259-66.

53. Goddard AD, Dowd P, Chernausek S, Geffner M, Gertner J, Hintz R, et al. Partial growth-hormone insensitivity: the role of growth-hormone receptor mutations in idiopathic short stature. The Journal of pediatrics. 1997;131(1 Pt 2):S51-5.

54. Meacham LR, Brown MR, Murphy TL, Keret R, Silbergeld A, Laron Z, et al. Characterization of a noncontiguous gene deletion of the growth hormone receptor in Laron's syndrome. The Journal of clinical endocrinology and metabolism. 1993;77(5):1379-83.

55. Godowski PJ, Leung DW, Meacham LR, Galgani JP, Hellmiss R, Keret R, et al. Characterization of the human growth hormone receptor gene and demonstration of a partial gene deletion in two patients with Laron-type dwarfism. Proceedings of the National Academy of Sciences of the United States of America. 1989;86(20):8083-7.

56. Yamamoto H, Kouhara H, Iida K, Chihara K, Kasayama S. A novel growth hormone receptor gene deletion mutation in a patient with primary growth hormone insensitivity syndrome (Laron syndrome). Growth hormone & IGF research : official journal of the Growth Hormone Research Society and the International IGF Research Society. 2008;18(2):136-42.
